# Supplementary figures and images for: Nondisruptive inducible labeling of ER-membrane contact sites using the Lamin B receptor
Source: PLoS Biol. 2025 Jul 10;23(7):e3003249. doi: 10.1371/journal.pbio.3003249 (PMC12244557; doi:10.1371/journal.pbio.3003249)

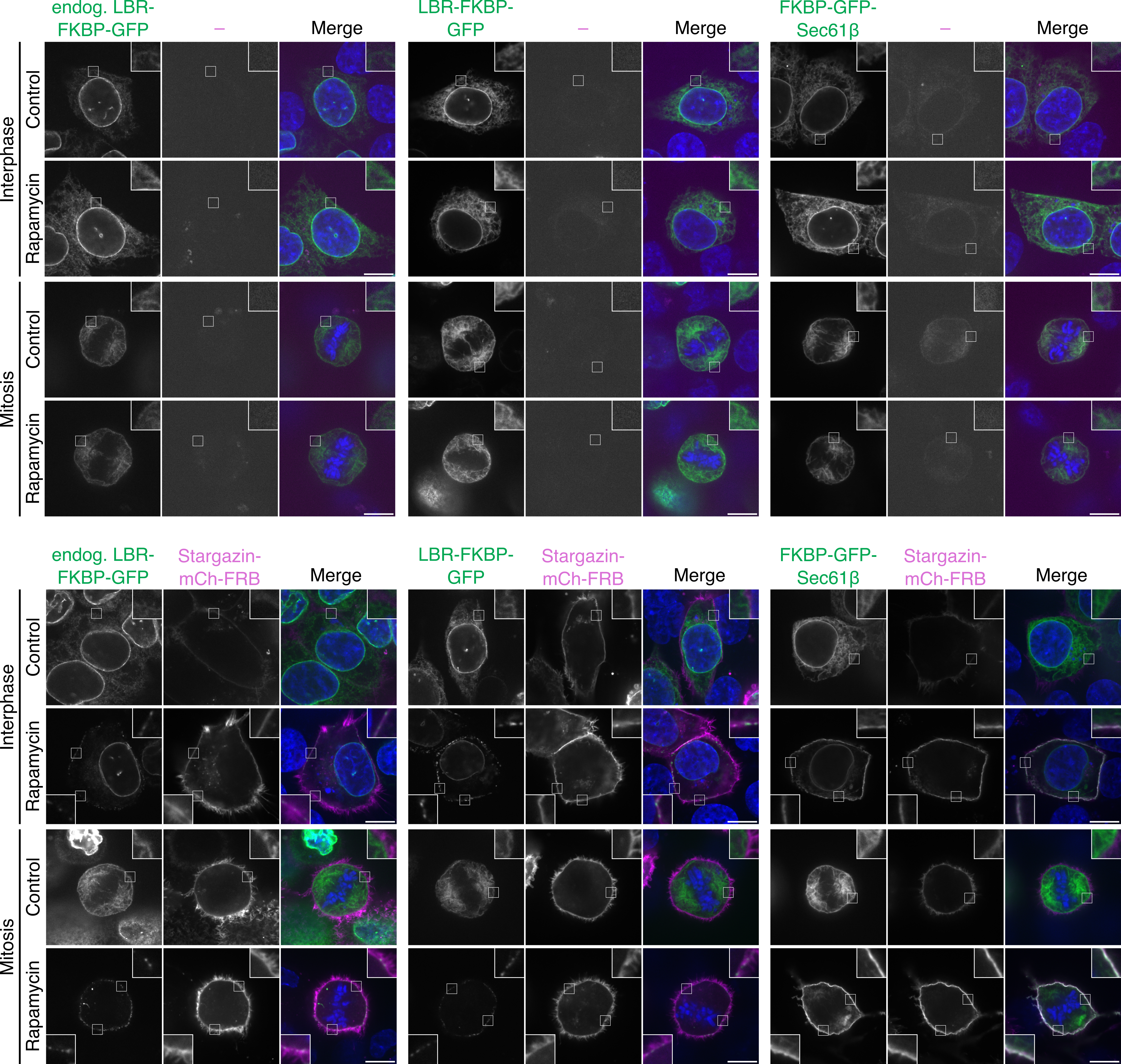

Supplement: S1 Fig — Example micrographs of HCT116 wild-type cells transiently expressing LBR-FKBP-GFP or FKBP-GFP-Sec61β (green) and HCT116 LBR-FKBP-GFP CRISPR knock-in cells, transiently expressing Stargazin-mCherry-FRB (magenta) as indicated and stained with DAPI (blue). Relocalized samples were treated with rapamycin (200 nM) for 30 min before fixation. Control samples were not treated with rapamycin. Scale bars, 10 µm; Insets, 3× expansion of ROI. (TIFF) [file pbio.3003249.s001.tiff]

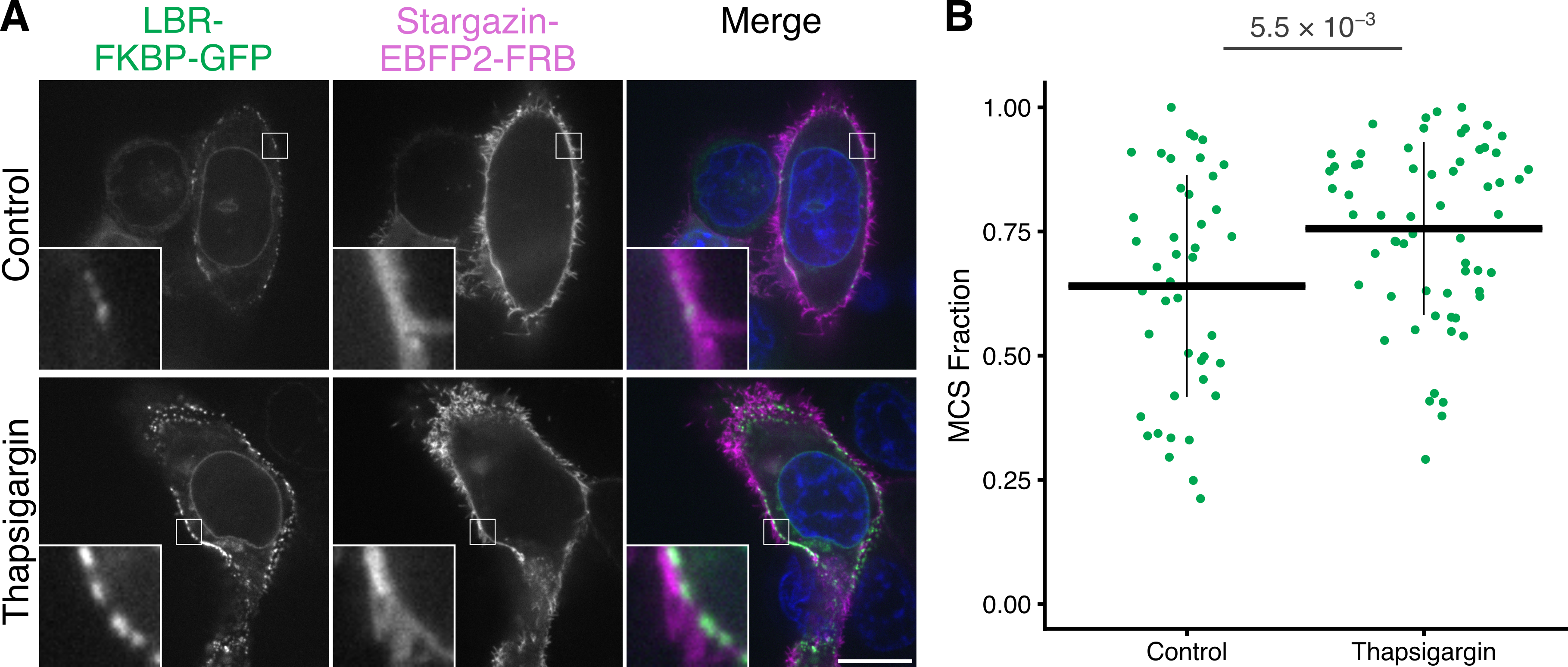

Supplement: S5 Fig — (A) Typical confocal images of HCT116 cells co-expressing LBR-FKBP-GFP (green), Stargazin-EBFP2-FRB (magenta), with SiR-DNA (blue in merge) to detect DNA. Cells were treated with thapsigargin (1 µM, 20 min) or not, as indicated, before relocalization was induced with rapamycin (200 nM). Scale bar, 10 µm; Insets, 4× expansion of ROI. (B) Plot to show the MCS fraction of the plasma membrane profile. Spot, cells; bars, mean ± sd. P-value, Student’s t test. The individual values for panel B are available at https://doi.org/10.5281/zenodo.15582238. (TIFF) [file pbio.3003249.s005.tiff]

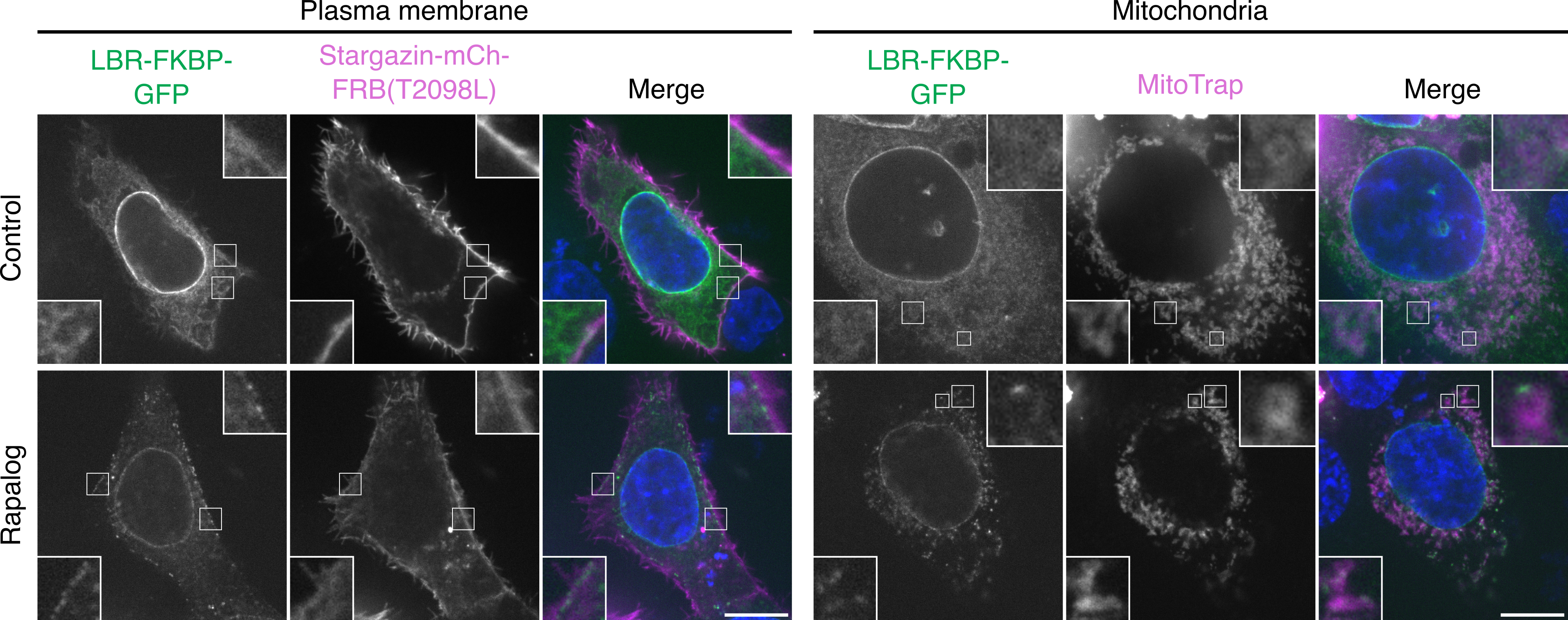

Supplement: S7 Fig — Single slices from z-stacks of fixed HCT116 cells co-expressing LBR-FKBP-GFP (green) and Stargazin-mCherry-FRB(T2098L) or MitoTrap (Mito-mCherry-FRB[T2098L]) (magenta) and stained with DAPI (blue). Where indicated, samples were treated with rapalog (5 µM) for 30 min prior to fixation. Scale bars, 10 µm; Insets, 6× expansion of smaller ROI or 3× expansion of larger ROI. (TIFF) [file pbio.3003249.s007.tiff]

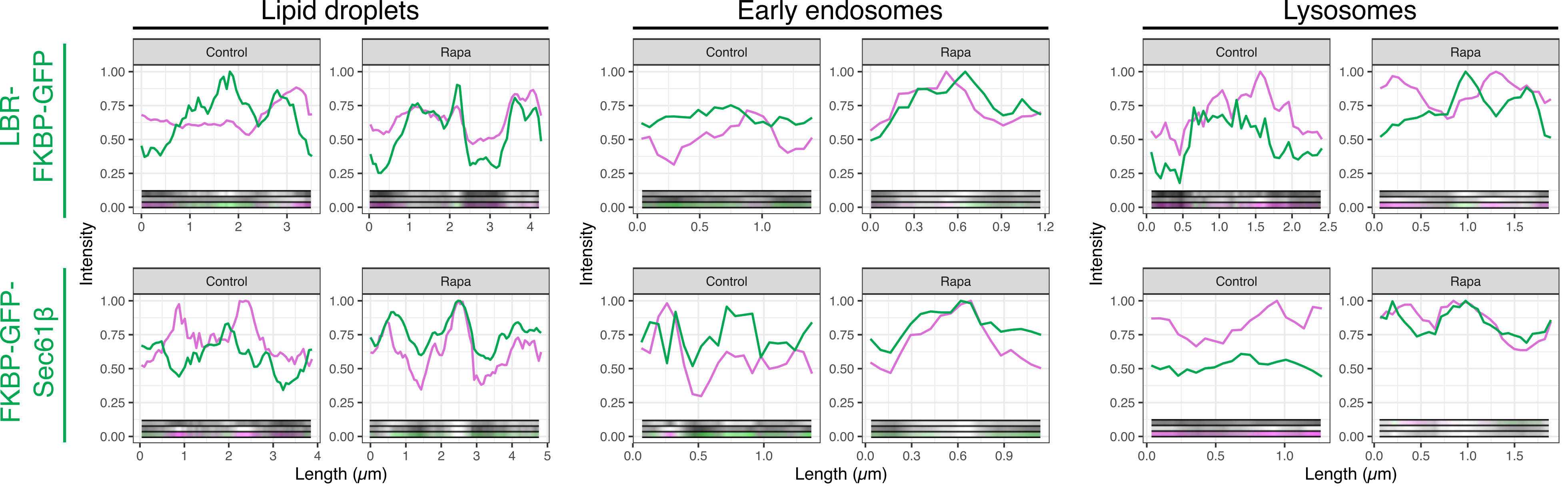

Supplement: S9 Fig — Line profiles corresponding to the cells shown in Fig 5A. Plots show the intensity of LBR-FKBP-GFP or FKBP-GFP-Sec61bβ (green) and anchor protein (magenta) signal measured around the perimeter of the structure. Anchor proteins are as follows: FRB-mCherry-PLIN3 (lipid droplets), FRB-mCherry-EEA1 (early endosomes), or LAMP1-mCherry-FRB (lysosomes). Insets show the line profile images. The individual values are available at https://doi.org/10.5281/zenodo.15582238. (TIFF) [file pbio.3003249.s009.tiff]

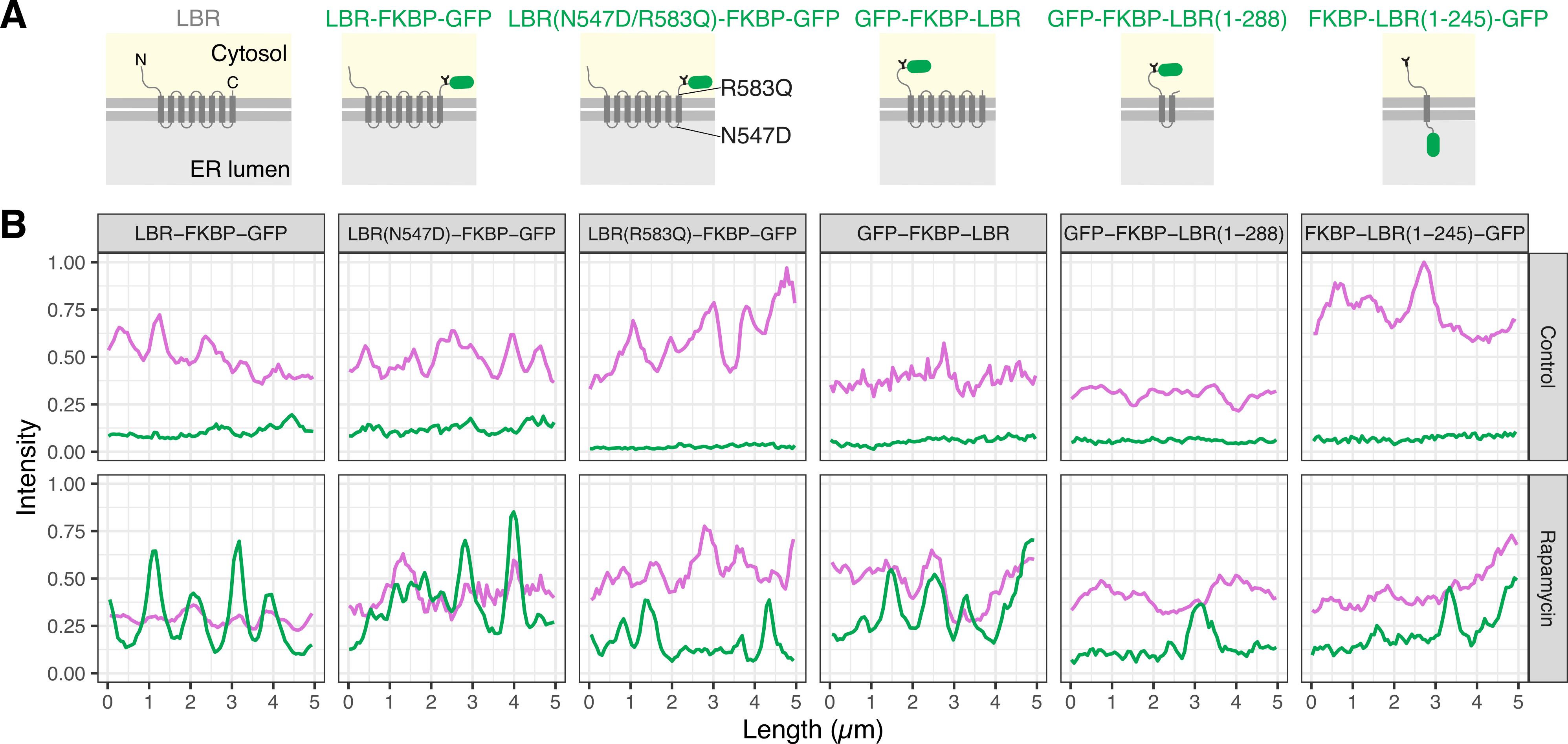

Supplement: S11 Fig — Line profiles corresponding to the cells shown in Fig 6. Plots show the intensity of FKBP-GFP-tagged LBR constructs (green) and Stargazin-mCherry-FRB (magenta) signal measured at the plasma membrane of mitotic cells. The individual values are available at https://doi.org/10.5281/zenodo.15582238. (TIFF) [file pbio.3003249.s011.tiff]

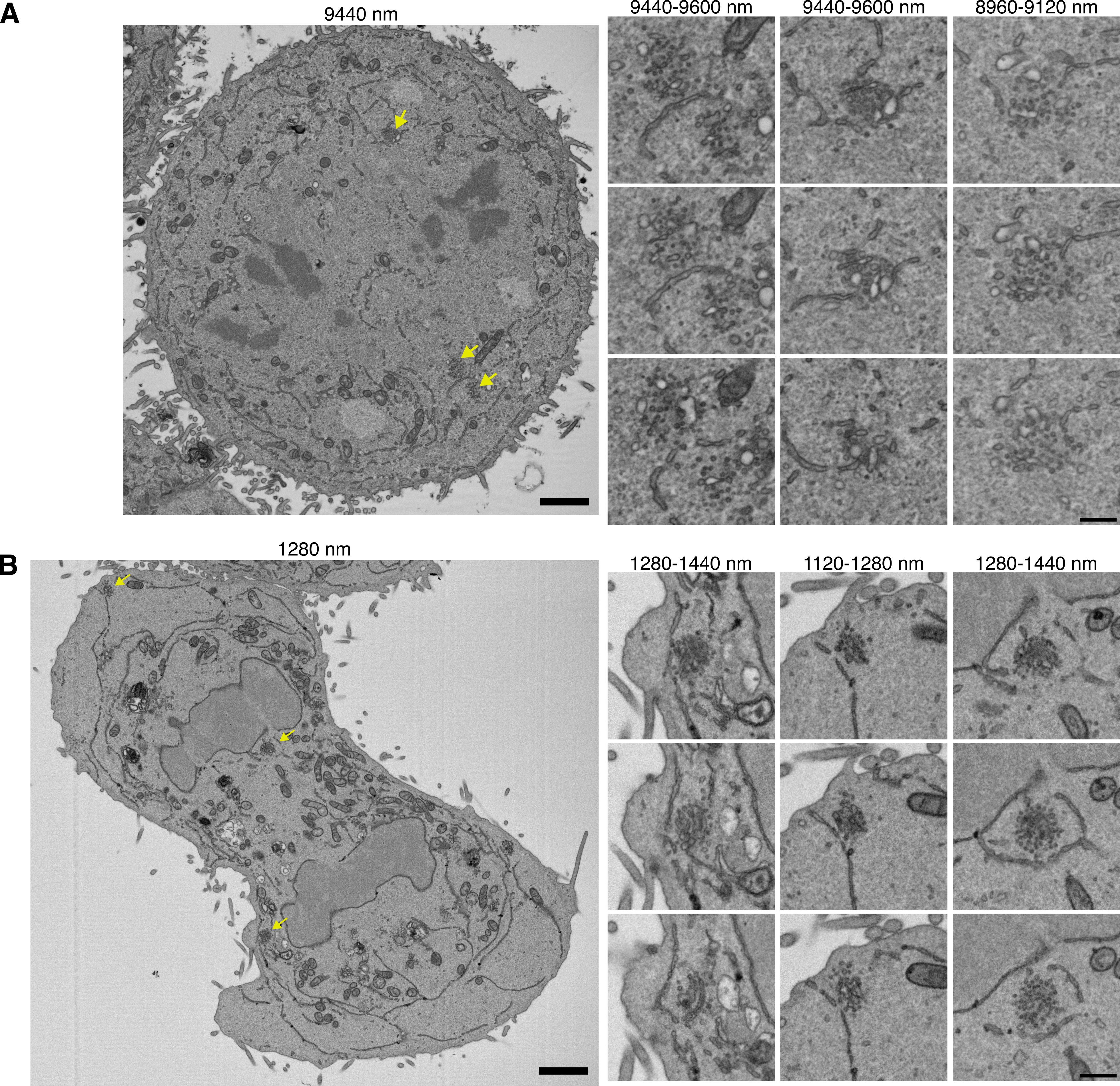

Supplement: S13 Fig — Single slices of metaphase (A) and telophase (B) HCT116 LBR-FKBP-GFP CRISPR knock-in cell SBF-SEM datasets are shown. Depth of each slice within the dataset (nm) is indicated. Example Golgi clusters are shown by yellow arrows on the full slice image. Three sequential slices of these regions (3 × expansion) are shown beside. Scale bars, 2 and 0.5 µm on zoom region. (TIFF) [file pbio.3003249.s013.tiff]

S2C

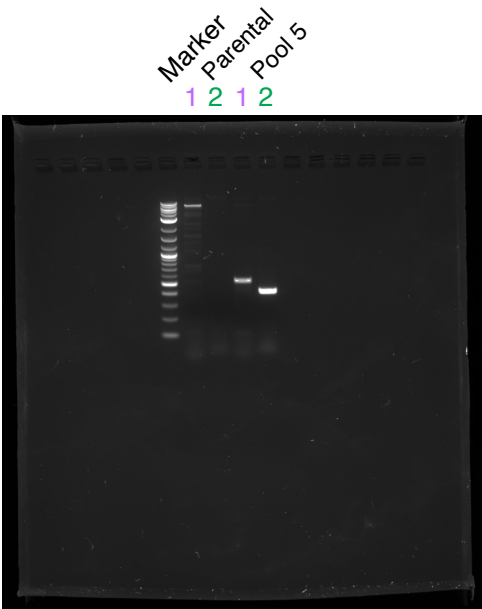

S2D

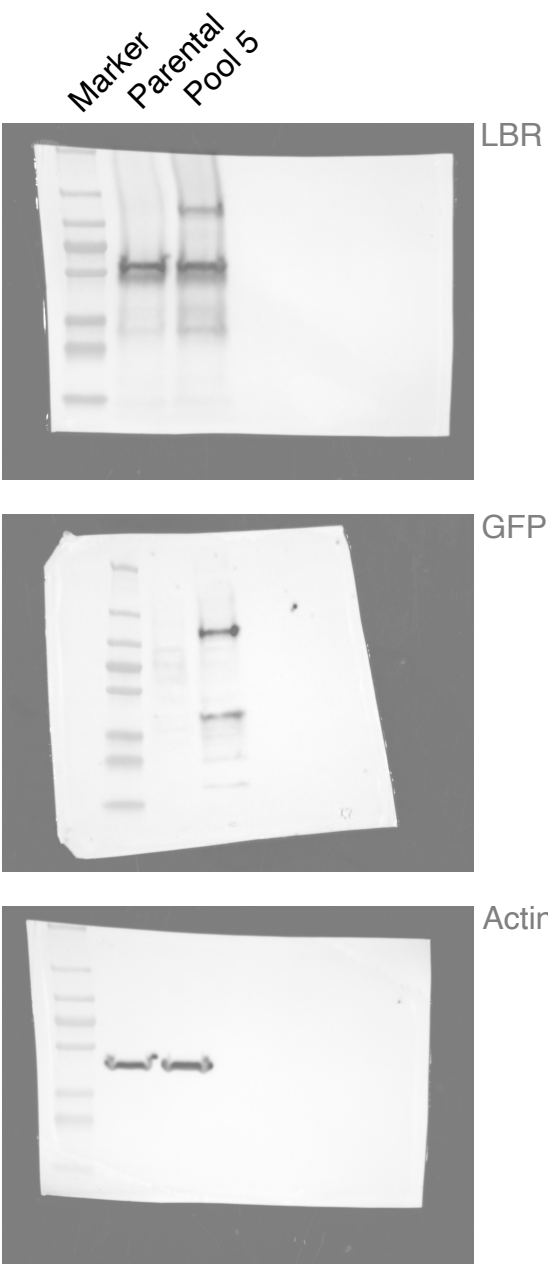

Supplement: S1 Raw Images — (PDF) [file pbio.3003249.s024.pdf]
