# Supplementary material for: Nondisruptive inducible labeling of ER-membrane contact sites using the Lamin B receptor
Source: PLoS Biol. 2025 Jul 10;23(7):e3003249. doi: 10.1371/journal.pbio.3003249 (PMC12244557; doi:10.1371/journal.pbio.3003249)
Supplement: Attachment — Submitted filename: ResponseToReviewers.pdf [file pbio.3003249.s026.pdf]

Reviewer 1

We thank the Reviewer for their time and valuable comments on our manuscript.

> Labelling membrane contact sites is a challenge. While several methods have been proposed, they suffer from either low signal to noise ratio or from artificial stitching of the organelles together. One popular approach is to direct a dimerizable fluorescent fusion protein to a given organelle and its dimeric partner in another organelle. The dimerization can only happen at contact sites between both organelles, and therefore traps the fluorescent protein there. The problem with this approach is that the force necessary to trap the fusion protein also contributes to the tethering force between the two membranes, leading to disturbance of the contact sites. This study proposes a variation on the theme using a fusion to ER-directed lamin B receptor and a drug-inducible dimerization module (FKBP-FRB) to trap GFP at various contact sites upon rapamycin treatment. The study shows with convincing analyses that this approach outperforms a similar approach based on ER-directed Sec61beta. They observe localization of the reporter to much more discrete puncta using LBR compared to Sec61B, and a tomographic analysis shows that the system does not detectably disrupt ER-mitochondria contacts when used with a mitochondrially targeted dimerizer. If the data show that the method provides adequate results in this configuration in practice, there is little theoretical explanation as to why this should be the case, and therefore it is hard to know how generalizable the approach is. As said before, the force that keep the reporter at the contact site contributes necessarily to the membrane tethering, and therefore disturbs the contact site. The amount of disturbance to any contact site is therefore dependent on 1/ the force of the dimerization interface, and 2/ the number of dimerizing molecules that localize there (a third parameter that could theoretically influence artificial tethering is the linker length, but it can be omitted since the authors "observed that various anchor proteins can be used successfully, which argues against the idea that physical spacing can explain the labeling behaviour"). Because both the sec61a and LBR-based approach use the same dimerizer, parameter 1 must be identical; and therefore, the difference in behaviour of the two system must be due to parameter 2 - protein amount. It seems trivial that decreasing the amount of protein by any possible mean is a good mean to decrease artefacts. Of course, decreasing protein amounts will decrease the signal-to-noise ratio to the point that the reporter might not be usable. So, it appears that the authors are in a "goldilocks" situation with their cell line and their crispr knock-in clone when assessing ER-mitochondria and ER-PM contacts, but whether this is true in other conditions, other expression vectors, other cell lines remains unclear.

We thank the Reviewer for this perspective. The purpose of our paper is two-fold: (i) to show that LBR can be used to label ER membrane contacts and (ii) to report the existence of Golgi-ER contacts during mitosis. A secondary aspect to the paper is *why* LBR can be used in this way. The answer to this question doesn't really matter for the two central messages of our paper.

The Reviewer raises a great point about whether the apparent "intrinsic property" of LBR is simply its expression level. We clarify why we don't think this is the case below.

> The authors appear to believe that an intrinsic "property of MCS targeting is contained in the N-terminal region and first TM domain" of LBR. This Reviewer concludes that the authors believe their results are explained by more than the protein amount parameter. However, they do not discuss what this property can be and how it may affect MCS targeting. In the absence of an explanation for this intrinsic "property of MCS targeting", and in the absence of a thorough demonstration of the contrary, we can only default to the most parsimonious explanation, i.e. that the differences are in fact due to different protein abundance at the MCS.
> In conclusion, it appears that with this system, the authors have hit on a goldilocks amount of protein expression to obtain detectable signals with minimal disturbance of the contact sites they could investigate (ER-mitochondria and ER-PM) in HCT116 cells. Whether LBR indeed has got inrinsic "MCS targeting properties" and whether this approach can be extended beyond this cell line is

> unclear.

Firstly, we have added data to show that LaBeRling can be used in RPE-1, Cos-7, and HeLa cells (Supplementary Figure S6). We show the highlighting of PM-ER, Mitochondria-ER and Golgi-ER MCSs in these three cell lines, to supplement the demonstration in HCT116 cells and show that LaBeRling can be extended beyond HCT116 cells. Secondly, we analyzed the expression of LBR-FKBP-GFP and FKBP-GFP-Sec61β by western blotting (Supplementary Figure S11). It is true that the expression of LBR-FKBP-GFP is lower than that of FKBP-GFP-Sec61β, however the expression of the truncated LBR constructs is higher than that of Sec61β, and since these can label MCSs, it argues against the idea that labeling is solely due to lower ER expression. We know that overexpression of LBR-FKBP-GFP doesn't disrupt the contacts. However, the counter-view is that even under conditions of higher expression, because the nuclear envelope pool of LBR may be anchored, the ER pool could still be less that that of Sec61β. Thirdly, to tackle this, we reduced the expression of FKBP-GFP-Sec61β using either a PGK or a crippledCMV promoter. Despite the lower levels of Sec61β, distortion of PM-ER contacts was still seen (Supplementary Figure S12). These data argue against a goldilocks expression scenario.

The Reviewer's criticism – that we didn't nail what exactly is the molecular determinant in LBR – is a fair one. We considered culling this section from the paper, but we feel that ruling out sterol biosynthesis is important, and that our finding that the N-terminal region is required was interesting enough for inclusion. We still feel that intrinsic targeting properties for LBR is the most viable explanation. Indeed, a very recent paper (PMID: 39342616) indicates that LBR may actually organize mitochondrial-ER MCSs during mitosis by associating with the IP3 receptor in the ER and VDAC in the mitochondrial membrane. If this is true, then the molecular determinant could well be its association with the IP3 receptor. This possibility is now added to a reworked Discussion paragraph (see below).

> Major points:
> -The LBR-FKBP-GFP cell line is a CRISPR knock in but the how other fusion genes are introduced in cells is less clear. It appears to be by transient transfection. Because of the importance of protein expression levels, it is crucial to indicate which method was used (including plasmid amounts). It is possible that most of the difference observed herein is due to the different methods of transgene delivery.

Apologies that this was not clear – transient transfection was used throughout. We have edited the methods section to make this clear and provide details of the procedure:

> For transient transfection, Fugene-HD (Promega) was used according to the manufacturer's instructions. A total of $1\,\mu g$ DNA with $3\,\mu L$ reagent was used per well of a six-well plate. With the exception of LBR-FKBP-GFP expressed endogenously, the expression of all constructs was via transient transfection.

> -Since the authors believe that LBR has got intrinsic MCS targeting properties, that are necessary for the success of their approach, a section of the discussion should be devoted to discussing these.

It is difficult to completely rule out the Reviewer's argument that LBR is in a "goldilocks" expression state. We reiterate that the reason LBR can be used in this way doesn't matter for the two central messages of the paper. The third paragraph of the Discussion is devoted to discussing this issue.

Reviewer 2

We thank the Reviewer for the time they took to read and comment on our manuscript.

> The enormous interest in the field of membrane contact sites (MCS) in recent years has generated a need for tools to study their biology. The manuscript by Downie et al presents a novel method for labelling MCS that takes advantage of an intriguing property of the Lamin B Receptor (LBR) to specifically label ER contact sites following chemically induced heterodimerization with target membrane proteins on other organelles. Using this "LaBeRling" system, the authors are able to identify ER:Golgi MCS at different stages in the cell cycle. The manuscript is high quality and well written, but while the conclusions are supported by the data, the data sometimes feel a little preliminary and there are some additional issues with the study design. Harnessing the apparently unique property of LBR as a specific contact site label has the potential to advance available MCS analysis methodologies and provide a valuable resource for the community, but the use of FKBP/FRB may limit its potential. Areas of concern are detailed below.

We were very pleased that the Reviewer appreciated our system to label ER MCSs on-demand.

> General comments
> * Throughout the manuscript, the authors fail to demonstrate that induction of LBR relocalization does not cause any global effects on the ER. The ER should be co-stained. If the tagged constructs used for LaBeRling don't allow expression of additional fluorescent ER-localised proteins, then an ER tracer dye can be used for live cell imaging or antibody staining for fixed cells.

This comment suggests that the Reviewer has overlooked three pieces of evidence in our paper. First, the complete 3D reconstruction of ER networks by SBF-SEM in multiple cells with LaBeRling of mitochondria-ER MCSs vs controls (Figure 4). This exercise didn't reveal any gross changes in ER morphology and our analysis showed that the mitochondrial-ER MCSs do not change, which in itself is a reasonable approximation of ER morphology. Second, throughout the paper we compared LBR relocalization with that of Sec61β – which clearly does have global effects on the ER – and found no such changes. Third, we showed light microscopy data where the ER was labeled while LaBeRling occurred (now Supplementary Figure S3B). Nonetheless, we have now added more experimental data to address this concern.

We now show staining of the ER with PhenoVue Fluor 568–Concanavalin A in fixed cells where PM-ER MCSs have been LaBeRled (Figure S4). This demonstrates that the relocalized LBR-FKBP-GFP is in the ER at the plasma membrane but that the ER is not coating the plasma membrane. We also attempted doing this in live cells with ER Tracker dye (as suggested) but found that this dye strongly stains mitochondria in addition to the ER, making the ER difficult to visualize. The upshot of all of this work is that we detect no gross defects on ER morphology induced by LaBeRling.

> * A potential major shortcoming to the application of LaBeRling is its dependency on the mTORC1 inhibitor rapamycin to induce relocalisation of LBR, since as well as affecting organelle function, rapamycin at concentrations as low as 100nM (used here at 200nM) can itself influence MCS (eg, ER:mitochondria contact sites, PMID: 27808250), which raises a serious question mark over the validity of this system for MCS measurement.

This criticism is valid, although the effects of rapamycin that the Reviewer is referring to happen on a much longer time scale than LaBeRling. The cited paper shows disruption after 4 hours of treatment – a similar time to autophagy induction by rapamycin – whereas LaBeRling occurs within a minute; and our movies were typically up to 30 min duration. Nonetheless, other investigators may wish to do LaBeRling for longer times. Under these circumstances, they can switch to Rapalog AP21967 using anchors with a modified FRB domain (T2098L) mutation.

To demonstrate this point, we have added experimental data to show:

1. LaBeRling of PM-ER and mitochondria-ER MCSs with rapalog (Supplementary Figure S7)
2. Long-term LaBeRling of PM-ER MCSs with rapalog (Supplementary Figure S8)
3. No effect on cell migration or viability under conditions of long-term LaBeRling (up to 8.5 h) of PM-ER MCSs with rapalog (Supplementary Videos 6 and 7)

* Another potential major shortcoming to employing the FKBP/FRB system is reversibility which has not been demonstrated. Irreversible interactions diminish potential advantages of LaBeRling as a dynamic MCS labelling approach; rapamycin-induced LBR translocation are very likely to influence MCS over time. This has not been adequately explored.

It is true that the LaBeRling system described in this paper is essentially irreversible (because the T_{off} of rapamycin is 16 h). Our demonstration, that LBR can be used in a plug-and-play style to rapidly label many different contact types, including novel ones, is the focus of this paper. It was not about demonstrating reversibility. This is an important direction for future development of this tool, and we thank the Reviewer for raising this topic. The final sentence of the Discussion, highlights this point.

The other aspect of this comment is whether irreversibly labeling contacts is detrimental to the cell (also raised by Reviewer 3). As described above, this issue is now addressed by our demonstration of long-term LaBeRling of PM-ER MCSs with rapalog (Supplementary Figure S8) which indicates that the contacts are not grossly affected over time and that cells are still able to migrate when PM-ER MCSs are LaBeRled (Supplementary Videos 6 and 7).

We also note that in our movies showing LaBeRling of Mitochondria-ER MCSs, the mitochondria are as mobile as in the control conditions which indirectly suggests that the relocalization doesn't grossly affect their dynamics.

* To validate utility for labelling MCS, known MCS regulators should be used to manipulate the contact and effects measured by LaBeRling versus a known reproducibly quantitative method (eg EM or proximity ligation assay).

This was a great suggestion. We used thapsigargin treatment to expand PM-ER contact sites, as described in PMID: 24183667. Following thapsigargin treatment we could indeed see more extensive PM-ER MCSs with LaBeRling (Supplementary Figure S5). This new data has improved the paper by demonstrating further utility.

Specific/minor comments:
1. p11, The LBR knockin cells in Fig1C should be introduced in the text before comparing Stargazin-dCherry-FRB to SH4-FRB-EBFP2.

This part of the paper has been reworked in light of other additions, but now the knock-in cells are introduced in sequence.

2. Fig1H. ER staining is needed to establish if there is any ER remodelling associated with LBR clustering at the PM.

We now show staining with PhenoVue Fluor 568–Concanavalin A staining of ER in fixed cells where PM-ER MCSs have been LaBeRled (Figure S4C). This shows that the relocalized LBR-FKBP-GFP is in the ER at the plasma membrane but that the ER is not coating the plasma membrane. Similar results are seen when visualizing the ER protein mCherry-Sec61β in live cells (Supplementary Figure S3B).

* Fig 2. While MAPPER clusters clearly aren't increased with LBR+rapamycin, consistent with MCS not being expanded, in 2C they appear substantially reduced, especially in interphase. Since MAPPER targeting to the PM requires the Rit polybasic motif to bind plasma membrane phosphoinosi-

> tides PIP2 and PIP3 (PMID 17095657), both of which the Stargazin cytoplasmic domain also bind directly (PMID 20547132), it seems possible that MAPPER and Stargazin might be competing for PIP binding at the PM. In which case MAPPER does not seem like an appropriate approach to examine effects of LaBeRling on ER:PM contacts and potential effects of LBR recruitment to plasma membrane MCS should be explored by a different method (eg, EM).

We hadn't considered this possibility. In our experience, PIP2 binders are not sufficient to outcompete other binders in the context of clathrin-mediated endocytosis, but it could be a possibility at MCSs. We settled on MAPPER as the reporter for PM-ER contact sites because other markers were unreliable in our hands. The experiments in Figure 2 involved full 3D reconstruction of cells at several timepoints and therefore suffered from photobleaching, despite our best efforts (noticeable in Figure 2A). This is the more likely explanation for the slight reduction seen, as we used the same segmentation approach for the whole movie. Even if there were competition, we would have found evidence for LBR clusters that were MAPPER negative, and we didn't (Figure 2C). For these reasons, we didn't embark on an EM study to look further at this point.

> 3. Fig 3A, The pMito staining too faint in the Sec61 + rapamycin panel, with no yellow visible in the merged image. This should be addressed, or different examples shown. The effect of rapamycin alone on mitochondrial dynamics and colocalisation of fluorescent proteins targeted to ER and mitochondria should also be shown.

We prepared the figure with the same scaling for all conditions, but this means some signals appear dim. In response to this comment (and Reviewer 4), we have adjusted the contrast on these images so that the anchor can be seen more clearly.

We have addressed the second point with two new pieces of experimental data. First, Supplementary Figure S1 shows rapamycin addition in cells expressing no anchor, and it shows no evidence for mitochondrial localization. Second, we have added data to show LaBeRling of MCSs using rapalog rather than rapamycin (Supplementary Figure S7). The fact that this works argues against the possibility that the effect of rapamycin non-specifically causes co-localization.

> 4. p4 When discussing fig 3B, it is stated that LBR clusters are not an artefact of overexpression. The knockin cell lines are a great addition to the study, but it should be noted that the pMito-mCh-FRB is overexpressed which could affect mitochondria dynamics and interactions. It would be nice to see mitotracker and colocalisation with an ER marker with and without pMito-mCh-FRB expression (± rapamycin).

We have been using pMito-mCherry-FRB extensively in our lab for about 14 years and in 9 papers. In our recent work we used expression of this plasmid to map the mitochondrial network (PMID: 37857498), so we're pretty confident that expression doesn't affect mitochondrial dynamics *per se*. This is not an 'argument from authority', high levels of expression *are* deterimental, it's just that with experience, we know to select lower expressing cells for our work, and had overlooked this issue. We have now added a note about this important point – which applies to all the anchors used – to the Methods section.

> 5. Figure 3B It appears that the green signal is increasing with time suggesting that LaBeRling might be stabilising/increasing the contact. Quantitation of LBR-FKB-GFP signal at puncta over time is needed and effect on global ER organisation needs to be shown. It would be nice to also show an interphase example. In the legend, Time is given in mm - should this be min?

It is not the case that the green signal increases over time. These experiments suffered from photobleaching due to the capture of z-stacks over time. This can be seen by comparing the 9 min frame to the 7 minute frame. So, while there is an initial increase when the signal of LBR-FKBP-GFP concentrates upon LaBeRling, the green signal weakens with the bleach. We struggled to quantify the fluorescence over time in the way that we had done in Figure 1H,I, because tracking the contacts frame-to-frame was chal-

lenging in 3D with the lower frame rate (required to minimize bleach) and considerable movement of the mitochondria.

In Figure 4, we quantify the size of thousands on mitochondria-ER contacts so we feel that the issue of whether LaBeRling stabilizes contacts is answered adequately here. The Reviewer's point that Figure 3 lacks quantification is a good one, and we have now addressed this point (also raised by Reviewer 4) by adding quantification of the fraction of mitochondrial profile that has a green signal (Figure 3D). This analysis shows nicely that the Sec61β label is far more extensive that the discrete contacts highlighted by LaBeRling.

Time is indeed in minutes and not millimetres... we have corrected the legend.

> 6. Figure 4, The duration of rapamycin treatment is important here and should be included in the legend. The depth/volume of SBF-SEM used for the 4A inference panel should be included in the legend. 4B, automated contact detection from EM images is a nice development/advance for MCS analysis! 4C should be reorganised to include an overlay to aid visualisation of any differences that occur after rapamycin. In 4D, in contrast to the text, the ER-mitochondria contact with larger distances (>60nm) clearly appear increased. Either this should be acknowledged/discussed (would there be increased closer contacts at later time points?) or, since MCS are often defined as being regions where membranes are 5-50nm apart, the contacts>60nm could potentially be excluded?

We were pleased the Reviewer liked our pipeline for detecting MCSs. The Figure has now been reorganized as suggested. We agree that only distances up to 50 nm are relevant. Beyond this distance, we are picking up nearby ER which is not part of the contact, so we have now capped the analysis at 50 nm. We have included contour plots to allow comparison, as suggested (this was a great idea). Finally, we have added the duration (30 min) and the size of the subvolume (48 slices) to the legend of Figure 4.

> 7. Figure 5, It's hard to see any difference between LBR and Sec61 recruitment to lipid droplets, the MCS appear just as extended after rapamycin with LBR. The selected examples for early endosomes look quite nice but may not be representative since bright EEA1 signal is completely surrounded by LBR. From the line plots there seems very little difference between LBR and Sec61 after rapamycin with lipid droplets or early endosomes - could quantitation of extent of colocalisation of lipid droplets/ early endosomes/lysosomes with LBR v Sec61 be shown as well? MCS quantitation by EM or proximity ligation assay, (eg with Duolink far red) ± rapa is needed to confirm if MCS extent is altered for all of these (looks increased for lipid droplets and early endosomes). Also lysosome signal looks quite different ± rapamycin.

We thank the Reviewer for raising this point. Our analysis was not very rigorous here and the Reviewer is right that the images and line profiles for lipid droplets are very similar between LBR and Sec61β. We have now improved the quantification by measuring the MCS Fraction (the fraction of an organelle profile that has LBR or Sec61β signal) at many examples of each of these contact types. For early endosomes and lysosomes we can see that the Sec61β coverage is more extensive than for LBR. However, for lipid droplets we do not detect a difference between the two. The Results section has been updated accordingly.

> 8. Fig 6 Not strictly necessary but it would have been nice to confirm the role of LBR N-Term by truncating the N-term with the FKBP-GFP on the C-term (LBR-FKBP-GFP).

Thank you for this comment. We generated a couple of N-terminal deletion constructs, but found that they exited the ER and were localized in the Golgi and small vesicles, which prevented their analysis.

> 9. Fig 7A Giantin signal in prometaphase and metaphase is very faint in Sec61+mScr panels. Need some form of quantitation and more/different examples. In 7C is this cell expressing the LaBeRling constructs? If so, this should be made clear in the legend and if not, it would be good to see effect of induced LBR label on the Golgi by EM (using CLEM to identify FRB-mCh-Giantin-expressing cells).

We have reworked this figure to incorporate several suggestions including making the ROI zooms larger and switching to a magenta-green color scheme. We took on board the request to provide some form of quantitation. We have used a simple colocalization analysis (Pearson's correlation coefficient) to look at the extent of colocalization between the Golgi anchor and our FKBP-tagged protein (LBR or Sec61β). We used this method rather than the MCS Fraction approach because using line profiles for the mitotic Golgi fragments was not possible. This suggestion (also raised by Reviewer 4 point 5) has improved the figure and strengthened our conclusions about Golgi-ER MCS LaBeRling.

We also provide more examples of the LaBeRling of this contact type in RPE-1, Cos-7 and HeLa cells (Supplementary Figure S6).

> 10. A relevant paper has recently been published (presumably since submission of this manuscript) which should be cited (PMID 38949658), as should reference to the widely used SPLICS approach (eg, PMID: 34686857).

We now reference these papers in the Introduction.

Reviewer 3

We thank the Reviewer for their helpful comments on our paper.

> I appreciate that a great deal of work has gone into this study, and fundamentally I am convinced that this approach successfully labels at least a subset of contact sites. However, I'm afraid that I have some significant concerns that need addressing prior to publication. These concerns, detailed below, revolve around incomplete comparisons with other similar approaches (e.g. split-FAST), incomplete controls on LBR expression levels, and a lack of adequate experimental evidence demonstrating the efficacy of LaBeRling following known MCS perturbations. These experiments are particularly important given that the mechanism by which the method described in this paper works, is unknown. While this is not in of itself a lethal problem, for this tool to be trusted, adopted and widely used by the community, a higher standard of evidence for its proper working is currently required.

We were really pleased that the Reviewer was convinced – albeit with some reservations – that our approach successfully labels contact sites.

> Major:
> 1) In the introduction, when discussing current techniques to label contact sites the authors appear to have overlooked split-FAST (link). This approach shares several of the purported advantages of LaBerRling (e.g. inducibility, unperturbing to MCS) but also, advantageously over LaBerRling, is reversible. The authors should both cite this work and address potential advantages/disadvantages versus LaBerRling.

We were aware of the split-FAST technology, but we had missed its application to contact sites ("PRINCESS" and "FABCON" methods). We now reference both of these papers in the Introduction.

There are advantages and disadvantages to all methods. We note the benefit of reversibility of split-FAST. We would counter that while this is an advantage, a downside of split-FAST is that it is proprietary tech. Our method doesn't rely on purchase of materials from "The Twinkle Factory". Researchers can grab our constructs off Addgene, get hold of rapamycin from any supplier, and they are good to go. Since we haven't used split-FAST we are not in a position to compare it with our method directly.

> 2) While LaBerRling is inducible, it is not reversible. All of the experiments in this paper seem to be conducted at short time intervals following rapamycin induction. For a new method such as this, it is important to know longer term potential effects on perturbing contact sites. What if the experimenter was to visualise contact sites over 2 hours? 4 hours? Particularly given the lack of reversibility in this system, a time series should be included.

As the Reviewer notes, our intention is for the method to be used short-term, but we were happy to investigate this point in case users wondered if LaBeRling can be used on a longer time scale. Due to the issues with long-term application of rapamycin, we show that rapalog AP21967 can be used as an alternative with the T2098L mutation in FRB (Supplementary Figure S7). Using this regime we show:

1. Long-term LaBeRling of PM-ER MCSs with rapalog (Supplementary Figure S8)
2. No effect on cell migration or viability under conditions of long-term LaBeRling (up to 8.5 h) of PM-ER MCSs with rapalog (Supplementary Videos 6 and 7)

> 3) How does LBR expression level affect its ability to label contact sites? The authors indirectly provide a comparison here between endogenously tagged LBR and overexpressed LBR but this should be tested more thoroughly. Is it possible that LBR simply works well to label contact sites because it is expressed at sufficiently low levels in the ER (with most of the protein being confined to the NE). These low LBR levels might not cause widespread 'wrapping' of the ER around the

> partner organelle, as seen with a more abundant protein like Sec61b. Unless the authors can show that their emerin, LAP2b and BAF controls also are expressed at similar levels in the ER (crucially not including the NE) I don't think this possibility can be excluded. Indeed Figure S3 does appear to show the formation of some puncta with these controls. An alternative approach might be to express Sec61-FKBP and LBR-FKBP at similar levels in the ER (again not including the NE) to ensure that the contact site labelling phenomenon, without organelle wrapping, is unique to LBR.

This point was also the main criticism raised by Reviewer 1. Firstly, we have now analyzed the expression of LBR-FKBP-GFP and FKBP-GFP-Sec61β by western blotting (Supplementary Figure S11). It is true that the expression of LBR-FKBP-GFP is lower than that of FKBP-GFP-Sec61β, however the expression of the truncated LBR constructs is higher than that of Sec61β, and since these can label MCSs, it argues against the idea that labeling is solely due to lower ER expression. We know that overexpression of LBR-FKBP-GFP doesn't disrupt the contacts (already shown in the paper). However, the counter-view is that even under conditions of higher expression, because the nuclear envelope pool of LBR may be anchored, the ER pool could still be less that that of Sec61β. Secondly, to tackle this, we reduced the expression of FKBP-GFP-Sec61β using either a PGK or a crippledCMV promoter. Despite the lower levels, distortion of PM-ER contacts was still seen (Supplementary Figure S12). These data argue against a scenario where LBR has a goldilocks expression level where it is low enough to be used in this way, and not high enough to distort contacts. Even if this is the case, we can say that the range of expression that allows LaBeRling is very wide. In indirect support of this, we have also added new data to demonstrate that LaBeRling is possible in three other cell lines (RPE-1, Cos-7 and HeLa), which further argues against the possibility that the application of LBR is limited by expression levels in one cell line.

The purpose of our paper is two-fold: (i) to show that LBR can be used to label ER MCSs and (ii) to report the existence of Golgi-ER contacts during mitosis. A secondary aspect to the paper is why LBR can be used in this way. The answer to this question doesn't really matter for the two central messages of our paper.

> 4) In Figure 1C the authors report that "there was minimal clustering of the plasma membrane anchor upon relocalization of LBR-FKBP-GFP". The authors speculate in the discussion that this might "mean that ER-MCSs are not truly bipartite and are perhaps governed by the ER". Given the relative endogenous abundance of ER tethers versus other organelle tethers, this would be surprising, and contradicts prior data suggesting that ER-MCS formation is NOT dictated by the ER, and instead by organellar partners (see link for example). Could the authors elaborate on this.

We were not precise enough here. We meant "governed by proteins on the ER side". This was implied by the preceding sentence, but the Reviewer is correct to pull us up that we seemed to be saying the ER itself was important. The Obara et al. paper nicely demonstrates this concept because the ALS mutation in VAPB which changes diffusivity, alters the mitochondria-ER MCS and supports our point. We now cite this paper in the Discussion.

> 5) Figure 2 uses a rapamycin-inducible system (MAPPER) as a positive control for LBR ER-PM contact sites. Are the authors concerned that MAPPER contact sites are somewhat artificial given the reasons they correctly raise about chemically inducible system? In any case there are plenty of ways of modulating the number of ER-PM contact sites (e.g. changing intracellular [Ca2+], modulating STIM1/Orai levels). The authors should perform such experiments and observe how these change LBR ER-PM contacts.

This is a misunderstanding: MAPPER is not rapamycin-inducible. In the original paper there is an FRB domain in the MAPPER construct (although this is not used for inducing labeling of contact sites) but the construct is constitutively at PM-ER MCSs. In our paper, we removed the FRB domain and replaced it with a similar structured protein (with PDB code 6DG5). We call our version of this construct mScarlet-I3-6DG5-MAPPER, and have made it available on Addgene since the original MAPPER construct is not in the public domain. We had to make this construct because we struggled to find antibodies or other constructs

that worked for specifically labeling MCSs (perhaps we were just unlucky).

Nonetheless, using something like MAPPER is a concern that we would alter PM-ER MCSs. We included the control of no MAPPER co-expression to assess the impact of expression.

The suggestion to modulate contacts is a good one. We used thapsigargin treatment to expand PM-ER contact sites, as described in PMID: 24183667 (the original MAPPER paper). Following thapsigargin treatment we could indeed see more extensive PM-ER MCSs with LaBeRling (Supplementary Figure S5). We think this is a nice addition to the paper which shows that we can detect changes in PM-ER MCSs with LaBeRling.

> 6) Figure 3 and 4: As with LBR ER-PM contact sites, there are plenty of ways to induce ER-Mito contact sites in cells (e.g. Tunicamycin, modulating PTPIP51, MFN1/2). The authors should see how these known parameters affect LBR ER-Mito contact sites.

With around 40 comments from 4 Reviewers to address, and the first author having limited time left on their contract; we down-prioritized this comment. We'd be happy for others to use LaBeRling to investigate manipulation of other ER MCs.

> 7) In Figure 5, the authors correctly state that the size of the organelles studied make distinguishing the MCS challenging. I'm afraid I don't find the displayed images or the lineplots here convincing. The FKBP-Sec61b and LBR-FKBP look similar after rapamycin addition. Again, I would look for other ways to increase/decrease these contact sites and repeat the experiment to provide a more convincing proof of concept.

We thank the Reviewer for raising this point (also raised by Reviewer 2). Our analysis was not very rigorous here. We have now improved the quantification by measuring the MCS Fraction (the fraction of an organelle profile that has LBR or Sec61β signal) at many examples of each of these contact types. For early endosomes and lysosomes we can see that the Sec61β coverage is more extensive than for LBR. However, for lipid droplets we do not detect a difference between the two. The Results section has been updated accordingly.

> 8) As above, for Figure 7, appropriate controls to modulate ER-Golgi contact site abundance should be included

This paper is the first report of Golgi-ER MCSs in mitosis. To our knowledge there is therefore no published example of a way to "modulate ER-Golgi contact site abundance" that we can use, and the Reviewer does not suggest one.

> Minor:
> 1) When describing Fig 1 the authors state that "Together these observations indicate that LBR-FKBPGFP clusters do not form at random locations at the plasma membrane.". It is not clear to me what is meant by 'random locations' or that the data in the figure suggest a 'random' distribution to be the case or otherwise.

The Reviewer is right: the meaning of this statement was unclear. We have now deleted it.

> 2) Controls without/before the addition of rapamycin should be included in the supplementary data for Figure 1

We have now included a figure which shows i) control vs rapamycin, ii) with and without expression of Stargazin-mCherry-FRB, iii) in interphase and mitosis, iv) for LBR-FKBP-GFP (endogenous or expressed) and FKBP-GFP-Sec61β (Supplementary Figure S1).

> 3) The lysosomes in Figure5 look very clustered in the perinuclear region. This doesn't look like a typical lysosomal distribution in healthy cells. Why is this the case?

Thank you for picking this up. We had selected some poor examples here and have now replaced with other images from the same experiment where the lysosomes are more distributed.

> 4) Typically, multipass TM domains are not able to properly access the single bilayer leaflet surrounding lipid droplets. Presumably LBR is able to do so in Figure5? Do you know how this is the case?

This is a misunderstanding. We use PLIN3 as the lipid droplet anchor which can access the leaflet. LBR or Sec61β are on the apposing ER membrane, not on the lipid droplet leaflet. When we induce relocalization, we recruit proteins on the ER-side to these areas of proximity. Similar labeling is seen with FABCON in a paper which was recently published (PMID: 38076863). We now cite this work as it provides better context for what we are doing with LaBeRling ER-LD MCSs.

> 5) Have the authors considered the possibility that LBR favours specific membrane curvature environments potentially shared between contact sites and the nuclear envelope?

This is a very interesting idea and it is one that we have discussed as a possibility within the lab. We have also wondered about diffusivity of LBR in the ER membrane affecting its ability to label contacts. Ultimately, we decided that these ideas are too speculative to include in the paper, since we have no data on this. Reviewer 1 argues that the only factor to consider is LBR density in the ER. In the paper we now tackle this factor experimentally, as well as the "molecular determinant" possibility. We have restrained ourselves from speculating about other properties of LBR, which include curvature preference.

> 6) Avoid Red/Green images throughout for colour blind compatibility

There was a rationale behind the color choices, and the manuscript was color-blind friendly. However, in light of this feedback (also from Reviewer 4) we reconsidered whether the mix-and-match was appropriate and we decided to switch everything to MGB.

Reviewer 4

We would like to thank the Reviewer for their time and thoughtful evaluation of our work.

> In a Methods and Resources style manuscript, Downie et al describe the fortuitous discovery that the Lamin B receptor (LBR) can be used as the ER anchor portion of an inducible membrane contact site (MCS) labeling construct. The development of visual markers of MCS has been a challenge for the MCS field. While many visual markers of MCSs have been described, the methods usually alter MCSs, such as stabilizing and/or expanding existing MCSs or creating artificial MCSs. Here, the authors nicely describe a new system in which LBR-FKBP serves as the ER anchor portion of a MCS labeling system they call LaBeRling. In the system, LBR-FKBP is expressed along with an FRB-tagged anchor protein on another organelle, and in the presence of rapamycin, the existing MCSs between the ER and the other organelle are labeled. The authors provide many experiments to demonstrate that the LaBeRling method can non-disruptively label MCSs. They also use the LaBeRling method to visualize ER-Golgi contacts in interphase and mitotic cells, the latter of which has not been visualized. The description of the ER-Golgi contacts provides an example of how the method can be used to uncover new biology. Overall, the described LaBeRling method will prove useful to the MCS field. However, given that this is the first description of the method, some experiments require more appropriate controls and quantification.

We were really pleased that the Reviewer thought that our new method will be useful. We address their comments below.

> Major comments:
> 1. There is a general lack of quantification of the data. In addition, it is not always clear how many biological replicates were done for all experiments shown and how many cells show the phenotype depicted in the representative image.

The Reviewer is presumably referring to Figures 5, 6 and 7 in the original manuscript, since Figures 1-4 had plenty of quantification. To address this criticism we have written new code to analyze the line profile data, so that we can show data from multiple cells. In the revised manuscript Figure 3D, and Figure 5B now show this measurement for mitochondria-, lipid droplet-, endosome- or lysosome-ER contacts. This allows direct comparison between LBR-FKBP-GFP and FKBP-GFP-Sec61β, which now allows us to make more definitive statements about differences between the two. The example line profiles, previously in the old Figure 5, are now moved to the supplementary information (Supplementary Figure S9). However, this methodology was not possible for Golgi-ER MCSs, so we have used a colocalization measurement to make a similar comparison (Figure 7). We have also added statements about the number of cells analyzed to the figure legends where they were missing. We thank the Reviewer for pushing us on these points because it has improved these parts of the paper.

> 2. I appreciate that there are limitations to space in each figure, but it is standard to show the non-induced (no rapamycin) condition as well as the induced (plus rapamycin) condition. While this is shown in some experiments, this control is missing from panel 1B. As Figure 1 begins to introduce the system, it would be a good control to include. This way readers can judge how much dimerization is occurring in the absence of the drug, which cannot be interpreted from the no Stargazin control.

We have now included a figure which shows i) control vs rapamycin, ii) with and without expression of Stargazin-mCherry-FRB, iii) in interphase and mitosis, iv) for LBR-FKBP-GFP (endogenous or expressed) and FKBP-GFP-Sec61β (Supplementary Figure S1).

> 3. In Figure 3A, why is there a decrease in the signal for mitochondria following rapamycin addition in cells expressing the Sec61 version of the tether? Also, for this figure, it would be better to use plus and minus rapamycin for labeling. Using "control" for the untreated is somewhat confusing because

> "control" in Figure 1 means the absence of the other half of the tether (Stargazin in that figure).

There is no decrease. Different cells are shown for control and rapamycin in Figure 3A, it just happens that the anchor expression is lower in these cells. We prepared the figure with the same scaling for all conditions, but this means some signals appear dim. In response to this comment (and Reviewer 2), we have adjusted the contrast on these images so that the anchor can be seen more clearly.

We have opted to change the labels on Figure 1B rather than change the labels for every subsequent Figure. Control/Rapa now consistently refers to application of the compound. Minus sign is used to indicate no expression.

> 4. That way Figure 3 is discussed in the text and displayed is somewhat confusing. Figure 3C is never called out in the results, and panels A and C are related but broken apart by panel B. Modification of the figure and associated results section would be beneficial for the reader.

Many thanks to the Reviewer for spotting this oversight. The results in the old Figure 3C did follow 3B, but this was not clear, and a call-out was missing. We have expanded this analysis to include many more fluorescent profiles to be able to say quantitatively that the coverage with Sec61β is higher than with LBR-FKBP-GFP. All panels of the figure are now referred to in the text.

> 5. Figure 7 uses LaBeRling to study ER-Golgi contact in interphase and mitotic cells and describes novel biology. While this is a nice proof of principle to demonstrate that the novel MCS marker can be used to uncover novel biology, the descriptive data need to be quantified for the data and conclusions based on these data to be included in the manuscript.
> 6. In addition, since Figure 7 describes novel biology, the appropriate controls of the experiment should be shown. It is unclear why mScarlet is used for Giantin lacking the FRB but mCherry is used for Giantin-FRB fusion. A more standard control would be mCherry and FRB-mCherry or mScarlet and FRB-mScarlet. Because different fluorophores are used, one cannot compare the control (no dimerization) with the experiment (dimerization). A more appropriate control would be to show the FRB-mCherry cells with and without rapamycin treatment.

We have used a simple colocalization analysis (Pearson's correlation coefficient) to look at the extent of colocalization between the Golgi anchor and our FKBP-tagged protein (LBR or Sec61β). We used this method rather than the MCS Fraction approach because using line profiles for the mitotic Golgi fragments was not possible. This suggestion (also raised by Reviewer 2 point 9) has improved the figure and strengthened our conclusions about Golgi-ER MCS LaBeRling.

We appreciate that mixing mScarlet and mCherry is not ideal, but there isn't really a basis for saying that this could affect the comparison: they are just fluorescent proteins after all. For this to be true, the mScarlet construct that lacks FRB should be capable of recruitment but that mScarlet interferes with that recruitment, which is rather far-fetched. To tackle the core concern here which is whether the Golgi-ER MCS LaBeRling is genuine, we now provide examples of Golgi-ER LaBeRling in RPE-1, Cos-7 and HeLa cells (Supplementary Figure S6). Importantly, here FRB-mCherry-Giantin(3131-3259) is expressed alongside LBR-FKBP-GFP, and the control of no treatment is shown alongside rapamycin treatment. This matches the referees request for more a appropriate control, and extends our observation beyond HCT116 cells, to three other cell lines.

> Additional comments:
> 7. In the introduction the authors state that "ER-MCSs were first observed in electron microscopy (EM) studies in fixed cells" and cite a 2019 review. While that is not an inaccurate reference, the original papers should be cited.

We have added references to four papers from 1950s and 1970s that describe contact sites using EM.

> 8. The authors should consider using color-blind-friendly choices for all their two-color images - green and magenta rather than green and red. Figure 2 is green/red, while Figure 4 is green/magenta.

There was a rationale behind the color choices, and the manuscript was color-blind friendly. However, in light of this feedback (also from Reviewer 3) we reconsidered whether the mix-and-match was appropriate and we decided to switch everything that was RGB to MGB.

> 9. I appreciate that regions of the fluorescence cell images are blown up in many figures for better visualization. If possible, it would be useful if they were expanded even just a little more to better see the important features of the protein localizations that authors are trying to highlight.

Thank you for this suggestion. We have expanded many of the zooms further so that they are external rather than inset in the image. See Figures 5, 7 and 8.
