# Supplementary material for: Nondisruptive inducible labeling of ER-membrane contact sites using the Lamin B receptor
Source: PLoS Biol. 2025 Jul 10;23(7):e3003249. doi: 10.1371/journal.pbio.3003249 (PMC12244557; doi:10.1371/journal.pbio.3003249)
Supplement: Attachment — Submitted filename: Plos_Biol_Rebuttal_Downie_et_al_Resub.pdf [file pbio.3003249.s028.pdf]

Reviewer 1

We thank the Reviewer for their time and valuable comments on our revised manuscript.

> In this revised manuscript, Downie et al. have done substantial work to address the reviewer's criticisms. The main criticism however remains: they propose a new method to label ER contact sites, but have no clue of how it works. Briefly, they use a rapamycin-dimerizable pair of proteins, one targeted to the ER, and one targeted to another organelle. They observe that using either full-length of fragments of LBR as ER-targeted moiety outperforms using sec61beta, and presumably (although not tested with the same rigor) other ER proteins. Yet, they cannot rationalize why.
>
> Major points:
> 1-Not being able to rationalize the data appears like a major shortcoming of the study. One could argue that, as long as it works, we do not have to care how it works. I disagree; to be useful, this method will need to be adaptable to the precise question that any user will want to put to it. Not knowing how it works makes it impossible how to adapt the method to ensure best results in a variety of scenarios. Perhaps, the authors could re-write their paper, not as a method, but as an observation: different ER-targeting moieties have different behaviours when they are artificially targeted to contact sites. Yet, with no physiological significance and no mechanistic insight, this observation appears orphaned. One way to obtain a beginning of a mechanistic insight could be to study in detail the (1-245) N terminal fragment that the authors claim is sufficient for the phenomenon. They could for instance exchange the N-terminal linker and the TM domains to figure which one is actually important for the phenomenon, and test possible models by mutagenesis.

We appreciate the Reviewer's view here but they are slightly overstating their point. It's not true that we have "no clue how it works". We have one protein in the ER (LBR) and another on the target membrane and we heterodimerize them to label the contact sites. This is essentially how it works. What is more mysterious is why LBR out performs other ER proteins for this task. We maintain that understanding why is not required to utilize LBR as a label. Our paper shows that LaBeRling does not distort MCSs and we go on to apply this to look at novel contact sites in mitosis. This is our preferred storyline for our paper and have not pivoted it as the Reviewer suggested.

In the revised manuscript, we address the concern about expression levels which the Reviewer has championed as the explanation for the difference (see below). We also provide a molecular information that the determinant for LaBeRling is in the N-terminal 245 residues of LBR and we exclude the possibility that lipid modification is the reason for the behavior.

> 2-I have raised the possibility that expression levels were responsible for the different behaviours of the tested constructs. The authors here provide additional data, where they express more or less of LBR-based constructs and sec61b-based constructs. Their conclusions is that it is the nature and not level of the protein that does the trick. Again, I beg to differ. In fig S12, the crippled CMV promoter driving low level of sec61-based construct solves pretty much all the problems of the sec61-based constructs. It does not increase the staining extent of the MAPPER reporter, indicating that it does not visibly increase the extent of ER-PM contacts. Also the protein itself colocalizes perfectly with MAPPER (the boxes that the authors highlight are, in that respect, not truly representative of the rest of the image), as seen with the LBR-based constructs. Therefore, while it likely does not explain 100% of the difference in the staining between LBR- and sec61-based reporter, expression level appears to be a major determinant of what does the trick.

We thank the Reviewer for their persistence on this point. Together with the comments from Reviewer 3 we realised that the work we had done was not convincing enough to answer this concern. Briefly, we have now quantified the ER fluorescence of FKBP-GFP-Sec61β under a CMV, PGK or crCMV promoter and compared this with LBR-FKBP-GFP expressed under a CMV promoter or expressed from the endogenous locus. We have also now carried out a 3D analysis to determine the size and density of the clusters that result following relocalization to Stargazin-mCherry-FRB. This shows that when the ER expression of

FKBP-GFP-Sec61β is equivalent to endogenously tagged LBR-FKBP-GFP, the cluster sizes are larger and the density of clusters is higher. Moreover, when LBR-FKBP-GFP levels in the ER are elevated (close to FKBP-GFP-Sec61β under a CMV promoter), the clusters are similar to the endogenously tagged version of LBR that has much lower expression. So the level of expression is not a major determinant of how LaBeRling works. We think that the resulting Figure (Supplementary Figure S12) now answers the point about expression raised by Reviewer 1 and Reviewer 3, and we thank Reviewer 1 for pushing us on this. Since the new Supplementary Figure S12 succinctly answers the expression question, we have removed the western blotting figure from the paper.

Two things to note: 1) we have renamed one of these promoters as crCMV since the original name of this promoter is somewhat offensive. 2) The Reviewer stated that relocalization of FKBP-GFP-Sec61β expressed under a crCMV "does not increase the staining extent of the MAPPER reporter, indicating that it does not visibly increase the extent of ER-PM contacts." We interpret this to be that MAPPER is marking the genuine MCS and that FKBP-GFP-Sec61β is gluing larger portions of ER to the PM. In the absence of MAPPER, using FKBP-GFP-Sec61β as a reporter of MCSs, investigators would be misled.

> 3-The authors have performed substantial work to answer the reviewers criticisms. One issue however is that these data are not quatified to the same standard. The data in their first version is precisely quantified and compared with each other. This care is commendable, but the need for such careful quantification also reflects the subtle quantitative differences (or absence thereof) reported here. The added new data is however not performed at the same standard. For instance, -the data on the differentially expressed constructs is not quantified and relies on visual inspection of micrographs, which as we have seen before, depends on the observer (my interpretation of the data on the crippled CMV promoter is opposite to the author's). -The additional cell lines that are used to show that the approach can be generalized are, again, not quantified. -Whether the single image of GFP-FKBP-LBR (Fig 6C) upon rapamycin looks more like LBR-FKBP-GFP (Fig 6B, S1) or like FKBP-GFP-Sec61 (Fig 1B, S1), is not obvious and left to the appreciation of the reader. My appreciation would again contradict the author's conclusions.

We have improved the quantification of the new data to the standard of the rest of the paper. The three points raised have been addressed by 1) the improved "expression" data where the ER expression is now precisely measured and 3D quantification of clusters from z-stacks is shown (discussed above, Fig S12); 2) addition of line profile data for the RPE-1, Cos-7 and HeLa cell lines for PM-, Mitochondria- and Golgi-ER MCSs LaBeRling (Fig S6B); 3) addition of line profile data for the LBR constructs (new Fig S11).

We have also improved quantification of long-term imaging data (Fig S8).

> 4-The Thapsigargin data are disappointing if the authors want to argue that their probe is sensitive to changes in ER-PM contacts. TG treatment is expected to multiply the extent of ER-PM contacts by 2 to 10 (link). The cited study utilizing MAPPER sees a 2-fold increase in MAPPER intensity. The ~20% increase reported here indicates that LabelER is not a great reporter for ER-PM contacts dynamics, or that the stimulus did not yield the expected increase in membrane apposition. It might be necessary to TG-treat cell in calcium-free medium if one wants to stimulate SOCE the 20-minutes necessary here. Anyways, to make a decisive point here, it is necessary to quantify the increased ER-PM apposition with another method (e.g. using the MAPPER, for which a good benchmark exists).

The Orci *et al*. study used EM to show a four-fold increase in the region of PM profiles that are in contact with ER following thapsigargin treatment. The MAPPER study on the other hand showed a 1.5-fold increase in spot density, with the size of the spots remaining unchanged.

In our previous experiments, we also only saw a small increase in PM-ER MCSs after LaBeRling. Prompted by this comment, we undertook substantial experimental work to try to see if we could detect larger changes. Briefly, we tried:

- HCT116 and HeLa cells
- Calcium-free media and regular media
- Different time points of thapsigargin

Despite this, we have not been able to detect a larger magnitude response with LaBeRling.

We confirmed using Fluo8-AM that our thapsigargin application was effective. So, we think that the original work may have overestimated the magnitude of increase, whereas our unbiased image analysis pipeline may be closer to measuring the true response. Importantly, in parallel we used MAPPER to look at changes using our image analysis pipeline. We consistently saw only a small response to thapsigargin application with MAPPER too (Reviewer Figure 1).

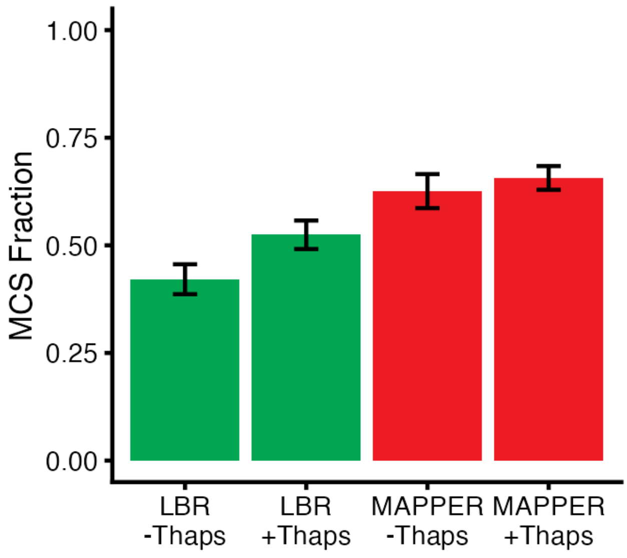

Figure 1: Effect of thapsigargin on PM-ER MCSs reported by LaBeRling or MAPPER. HeLa cells expressing LBR-FKBP-GFP and Stargazin-mCherry-FRB (green) or mScarlet-I3-6DG5-MAPPER (red). Cells were treated with thapsigargin ($1\,\mu M$, $20\,\text{min}$) as indicated.

Reviewer 2

We thank the Reviewer for the time they took to read and comment on our revised manuscript.

> The manuscript is much improved and many of my concerns have been addressed. However, the field has progressed during the revision period and in light of the recent report by Zhao et al (Cell Reports 2024) demonstrating a role for LBR as a tether connecting IP3R on the ER with VDAC2 on mitochondria to mediate mitochondrial calcium influx, the targeting of LBR to specific organelles seems likely to affect calcium homeostasis. This, coupled with intrinsic irreversibility, raises a question mark over the value of induced LBR relocalisation to the community as a tool to study MCS, which are often both important for and regulated by cellular calcium. Nevertheless a lot of work has gone into the study and some very nice data is presented. The revisions have improved the quality of the manuscript but I still have a few concerns:

We were pleased that the reviewer thought our revised paper was much improved and that we had addressed their concerns. The paper by Zhao *et al.* is interesting. We do not know if LaBeRling changes calcium homeostasis, although since LaBeRling only labels pre-existing MCSs we think the likelihood that it influences SOCE is lower than the Reviewer is proposing. Moreover, the paper reports a mitosis-specific association between LBR and VDAC2, which means that this concern might only be restricted to this phase of the cell cycle. We are pleased that the Reviewer recognizes that we are doing more in this paper than just showing that LBR can be used to label MCSs, for example the detection of mitochondrial-ER MCSs by machine learning in volumetric EM data and the description of Golgi-ER MCSs in mitosis are both novel aspects to our work.

> * Global effects of induction of LBR relocalisation on the ER: The authors claim that 3D-EM reconstruction of ER networks by SBF-SEM in multiple cells with LaBeRling of mitochondria- ER MCSs vs controls (Figure 4) didn't reveal any gross changes in ER morphology. The 3D-EM is a great addition to the paper, but I'm not seeing the analysis of ER morphology in Figure 4 - maybe I'm overlooking something? In the examples shown in A, there seems to be quite a striking difference in ER distribution in control v rapamycin-treated cells (SBF-SEM panels).

The distribution of ER is variable from cell-to-cell and the correlative SBF-SEM method is quite limited in the number of cells we can analyze practically. The example chosen in Fig 4 is our best example of correlation of light and electron microscopy images. The main message with this analysis is the examination mitochondria-ER MCSs at the ultrastructural level rather than the global ER distribution. To look at large-scale effects on the ER, we need to zoom out to light microscopy level. As the Reviewer notes below, the best way to assess the impact of LaBeRling on ER morphology is by observing the same cell before and after LaBeRling by fluorescence microscopy.

> Similarly, I understand that LBR relocalisation has been compared with that of Sec61β but would have liked to see that in the same cell - ie, does relocalisation of LBR to MCS following rapamycin affect ER morphology as visualised by other ER markers (in the same cell)? Fig S3B goes part way to address this, but without the no rapamycin control it's very hard to determine if ER distribution is affected. To me, the data shown appears to suggest that there is some rearrangement of the ER network with a high amount of Sec61 staining at the PM, but it's hard to tell if this is increased without seeing Sec61 distribution in the corresponding no rapamycin control cells. The new data in FigS4 is helpful but not many conclusions can be drawn from an image of a single cell, also with no controls.

The global effects on the ER following FKBP-GFP-Sec61β or LBR-FKP-GFP relocalization are night-and-day differences. One protein glues the ER to the target membrane and the other doesn't. This is what we refer to when we talk about LBR-FKP-GFP relocalization not causing gross changes in ER morphology. The Reviewer's concern here relates to whether there are more subtle changes in the ER network when we do LaBeRling. This is challenging to answer quantitatively because the ER is a highly dynamic network.

We can really only make qualitative statements here, but most immediate is that ER dynamics themselves do not seem to be obviously altered when we do LaBeRling.

To address this concern, we have:

- Added the control for the experiment where the ER is visualized with PhenoVue Fluor 568-ConA during LaBeRling to Fig S4A.
- Added an example where LaBeRling of PM-ER MCSs is done in cells also expressing mCherry-Sec61β (Fig S4B). This same cell analysis show before LaBeRling (the control condition) and after 7 minutes of LaBeRling.
- Added two new movies showing LBR-FKP-GFP and mCherry-Sec61β expressed with SH4-FRB-EBFP2 or not. Rapamycin is applied and the process of LaBeRling is seen together with the ER dynamics reported by mCherry-Sec61β (Videos SV2 and SV3).

> * Irreversibility: New data showing the effect of stabilising MCS (FigS8) is encouraging but it should be stated clearly that the LaBeRling system is irreversible rather than alluding to it vaguely in the final sentence (that contains a typo for membrane).

The heterodimerization of FKBP and FRB domains with rapamycin and derivatives has a long T_{off} but it is not irreversible. We have edited the final sentence to be clear that investigators who wish to repeatedly label MCSs would need to modify our method.

> 5. Figure 3B LBR puncta increase with time. The authors' assertion that green signal weakens over time as a result of photobleaching does not match what I see on the figure. Although the signal in the magnified area is reduced at 9 min, in the cell as a whole, the LBR puncta appear to be increased both in number and intensity. In contrast, the mitotrap signal has faded with photobleaching, so quantitation of LBR puncta:mito signal ratio would probably reveal a significant relative increase in LBR intensity. I would like to see quantitation of LBR puncta relative to the targeted organelle in large fields of view imaged at different (including longer) time-points after rapamycin. This doesn't need to be continuous imaging, or the same individual cell - snapshots in large numbers of cells at different timepoints could be used for quantitation. 3D adds a nice measurement of MCS induced by LBR v Sec61 but as far as I can tell only 3 cells were included in the analysis for control cells (4 for +Rapamycin) which, given the cell-to-cell variability in Figure 4D, is a very low number for meaningful quantitation. Also the scatter plots suggest increased "long" tethering (>30nm) of ER-mitochondria contacts, whereas a 20nm inter-organelle gap was shown to be optimal for IP3R-dependent Ca2+ transfer at the MCS, with reduced Ca2+ transfer at 30nm distnces (Dematteis et al, Commun Biol, 2024), suggesting that the LRN-FKBP-GFP construct may not be functional at the IP3R-VDAC2 complex. In terms of its potential utility as a MCS sensor, it would be important to know if it affects Ca2+ flux.

The main issue here is whether the LaBeRled MCSs get distorted over time. We previously added the requested long-term imaging experiments to address this point, but it was not quantified. We have now performed 3D analysis on many cells, after 2 h and 4 h of LaBeRling. We can correlate between these time points to look for distortion of MCSs in the same cell. Fig S8 now shows that the size of the cluster area does not increase between these two time points, so this argues that there is no distortion of MCSs even with very long LaBeRling times. Due to the method, our 3DEM analysis is limited by the number of cells. However, the number of mitochondrial-ER MCSs analysed is huge - many thousands. So the quantification is meaningful and the number of independent repeats is enough to assess reproducibility of our measurements. The scatter plots do not suggest any differences at any of the sizes we measured. Please see the contour overlays which show an overlapping distribution for control and LaBeRled condition.

Reviewer 3

We thank the Reviewer for their helpful comments on our revised paper.

> The authors have made some impressive improvements and meaningful experimental additions to the manuscript. I am convinced by most of their amendments with a few important exceptions below. Should they address these remaining concerns I would support publication of the work.

We were very pleased that the Reviewer was generally happy with our revision work.

> "We were aware of the split-FAST technology, but we had missed its application to contact sites ("PRINCESS" and "FAB-CON" methods). We now reference both of these papers in the Introduction. There are advantages and disadvantages to all methods. We note the benefit of reversibility of split-FAST. We would counter that while this is an advantage, a downside of split-FAST is that it is proprietary tech. Our method doesn't rely on purchase of materials from "The Twinkle Factory". Researchers can grab our constructs off Addgene, get hold of rapamycin from any supplier, and they are good to go. Since we haven't used split-FAST we are not in a position to compare it with our method directly."
>
> The authors have now included the appropriate references and their justification above is reasonable. However, this justification should be made clear in the manuscript e.g. these approaches rely on proprietary costly fluorophores. In its current writing, it seems as if the authors suggest that split-FAST also artificially increases contacts, which is not the case based on the data from the PRINCESS and FABCON papers.

We have added the following sentence to the Introduction. "Furthermore, inducible methods that are less disruptive rely on expensive proprietary reagents prompting the need for development of new labeling methods (García Casas et al., 2024; Tebo and Gautier, 2019)."

> "As the Reviewer notes, our intention is for the method to be used short-term, but we were happy to investigate this point in case users wondered if LaBeRling can be used on a longer time scale. Due to the issues with long-term application of rapamycin, we show that rapalog AP21967 can be used as an alternative with the T2098L mutation in FRB (Supplementary Figure S7). Using this regime we show:
> 1. Long-term LaBeRling of PM-ER MCSs with rapalog (Supplementary Figure S8)
> 2. No effect on cell migration or viability under conditions of long-term LaBeRling (up to 8.5 h) of PM-ER MCSs with rapalog (Supplementary Videos 6 and 7)"
>
> These are useful additions to the manuscript and although only demonstrated for one contact site, this seems like an adequate response.

We were very pleased that the Reviewer was happy with the work that we did to address their concern.

> "This point was also the main criticism raised by Reviewer 1. Firstly, we have now analyzed the expression of LBR-FKBP-GFP and FKBP-GFP-Sec61β by western blotting (Supplementary Figure S12). It is true that the expression of LBR-FKBP-GFP is lower than that of FKBP-GFP-Sec61β, however the expression of the truncated LBR constructs is higher than that of Sec61β, and since these can label MCSs, it argues against the idea that labeling is solely due to lower ER expression. We know that overexpression of LBRFKBP-GFP doesn't disrupt the contacts (already shown in the paper). However, the counter-view is that even under conditions of higher expression, because the nuclear envelope pool of LBR may be anchored, the ER pool could still be less that that of Sec61β. Secondly, to tackle this, we reduced the expression of FKBP-GFP-Sec61β using either a PGK or a crippledCMV promoter. Despite the lower levels, distortion of PM-ER contacts was still seen (Supplementary Figure S12). These data argue against a scenario where LBR has a goldilocks expression level where it is low enough to be used in this way, and not high enough to distort contacts. Even if this is the case, we can say that the range of expression that allows LaBeRling is very wide. In indirect support of this, we have also added new data to demonstrate that LaBeRling is possible in three other cell lines (RPE-1, Cos-7 and HeLa), which further argues against the possibility that the application of LBR is limited by expression levels in one cell line. The purpose of our paper is two-fold: (i) to show that LBR can be used to label ER MCSs and (ii) to report the existence of Golgi-ER contacts during mitosis. A secondary aspect to the paper is why LBR can be used in this way. The answer to

> this question doesn't really matter for the two central messages of our paper."
>
> I'm afraid I don't find this additional data very convincing. As the authors recognise, the critical issue here is the protein levels of ER localised LBR, not total LBR (thus the WB does not really help sine it is ER+NE LBR levels). Likewise, the authors do not provide evidence that the crippled CMV or PGK promoter reduce ER levels of Sec61 to comparable ER levels of LBR. I propose two solutions, one is to carefully quantitate the ER levels of LBR vs Sec61 using the microscopy data collected (given the FP is identical and effects of self-quenching/folding should be comparable). The alternative simpler solution is to state clearly in the manuscript that this may indeed be a goldilocks expression level phenomena but it still works! I would find either approach acceptable, although of course the former is more intellectually satisfying.

This feedback was very useful and we went with the Reviewer's first proposed solution. Briefly, we have quantified the ER fluorescence of FKBP-GFP-Sec61β under a CMV, PGK or crCMV promoter and compared to that of LBR-FKBP-GFP expressed under a CMV promoter or from the endogenous locus. We have also now carried out a 3D analysis to determine the size and density of the clusters that result following relocalization to Stargazin-mCherry-FRB. This analysis shows that when the ER expression of FKBP-GFP-Sec61β is equivalent to endogenously tagged LBR-FKBP-GFP (i.e. very low), the cluster sizes are larger and the density of clusters is higher than those LBR. Moreover, when LBR-FKBP-GFP levels in the ER as elevated (close to FKBP-GFP-Sec61β under a CMV promoter), the clusters are similar to the endogenously tagged version of LBR that has much lower expression. We think that the resulting Figure (Supplementary Figure S12) now answers the point about expression raised by Reviewer 1 and Reviewer 3, and we thank Reviewer 3 for pushing us on this. Finally, we took on board the criticism that the western blotting was not convincing and that it doesn't fully address the point. Since the new Supplementary Figure S12 succinctly answers the expression question, we have removed the western blotting figure from the paper.

Note, we have renamed one of these promoters as crCMV since the original name of this promoter is somewhat offensive.

> "We were not precise enough here. We meant "governed by proteins on the ER side". This was implied by the preceding sentence, but the Reviewer is correct to pull us up that we seemed to be saying the ER itself was important. The Obara et al. paper nicely demonstrates this concept because the ALS mutation in VAPB which changes diffusivity, alters the mitochondria-ER MCS and supports our point. We now cite this paper in the Discussion."
>
> Apologies if my point wasn't made clearly the first time. I understood the authors initial prose, but instead was surprised by the assertion that the ER tethering partner governs contact sites and that the other organellar tethering partner may be less important. Figure 3 from the Obara et al., manuscript suggests that overexpression of VAPB (the ER partner) has very little effect on ERMCS abundance, while overexpression of PTPIP51 dramatically increases contact sites. This has been shown with other similar systems, where the ER tether abundance seems to have minimal effect, while the abundance of FFAT containing organellar partners seems to have a dramatic effect. As such, I would caution the authors against interpreting that "ER-MCSs are not truly bipartite and are perhaps governed by the ER".

Apologies for our misunderstanding (a problem of the postcard nature of peer review). We were keen to simply interpret what we were seeing, but the Reviewer is correct that our statement was misleading. A key point of LaBeRling seems to be that we are populating pre-existing MCSs with LBR, rather than forming new *bona fide* MCSs. So the fact that LaBeRling is driven by LBR on the ER side, may not be relevant to ER-MCS formation. As the statement wasn't necessary for this paragraph, we have deleted it.

> "This is a misunderstanding: MAPPER is not rapamycin-inducible. In the original paper there is an FRB domain in the MAPPER construct (although this is not used for inducing labeling of contact sites) but the construct is constitutively at PM-ER MCSs. In our paper, we removed the FRB domain and replaced it with a similar structured protein (with PDB

code 6DG5). We call our version of this construct mScarlet-I3-6DG5-MAPPER, and have made it available on Addgene since the original MAPPER construct is not in the public domain. We had to make this construct because we struggled to find antibodies or other constructs that worked for specifically labeling MCSs (perhaps we were just unlucky).Nonetheless, using something like MAPPER is a concern that we would alter PM-ER MCSs. We included the control of no MAPPER co-expression to assess the impact of expression. The suggestion to modulate contacts is a good one. We used thapsigargin treatment to expand PMER contact sites, as described in PMID: 24183667 (the original MAPPER paper). Following thapsigargin treatment we could indeed see more extensive PM-ER MCSs with LaBeRling (Supplementary Figure S5). We think this is a nice addition to the paper which shows that we can detect changes in PM-ER MCSs with LaBeRling."

Thank you for clarifying this point and apologies for the misunderstanding. I agree that the thapsigargin experiment is a strong addition to the paper and my initial point has been well addressed.
All my remaining concerns have been sufficiently addressed.

We thank the reviewer for their positive assessment of the revision experiments.

Reviewer 4

We would like to thank the Reviewer for their time and thoughtful re-evaluation of our work.

> I thank the authors for their very thoughtful responses to the concerns raised. The authors have adequately addressed the concerns I had with the initial manuscript. They have strengthened the data to support that the LaBeRling method can be used to non-disruptively label ER MCSs in cells, which will prove useful to the MCS field.
> One minor comment is regarding the changes made to Figure 3A in response to comments that both I and Reviewer 2 made. Using different scaling on the images is acceptable. However, the authors should state in the legend or methods that different scaling was used between panels and direct comparisons of intensity cannot be made.

We have now added a note in the figure legend.
