# Supplementary material for: Nondisruptive inducible labeling of ER-membrane contact sites using the Lamin B receptor
Source: PLoS Biol. 2025 Jul 10;23(7):e3003249. doi: 10.1371/journal.pbio.3003249 (PMC12244557; doi:10.1371/journal.pbio.3003249)
Supplement: Attachment — Submitted filename: PBIOLOGY-D-24-03172R2_Rebuttal.pdf [file pbio.3003249.s029.pdf]

Reviewer 1

We thank the Reviewer for their time and comments on our revised manuscript.

> The amount of new work by the authors is highly commendable, and I don't think further work can help make their case.
> I unfortunatley remain unconvinced of the importance and usefulness of this tool.
> 1-Reducing the expression level appears to do most of the trick, even if not correlating with absolute amounts of protein (see point 2).

We are disappointed that Reviewer 1 remains unconvinced of the applications of our system. Our disappointment is natural given the amount of work we put in to investigate the Reviewer's idea that expression levels are the reason that LBR can be used to label contact sites. However, despite what we feel is strong evidence that this idea is not correct, the Reviewer remains unconvinced. We feel that addressing this point has improved our manuscript. Ultimately, we are happy for readers to make up their own minds about this issue and are happy that journal publishes the peer reviews and our responses to them, alongside the paper.

> Somehow, using the BFP- rather than mCherry-tagged Stargazin also helps(figure s12C). The sec61b foci of figure S12C (PGK and crCMV) appear more discrete than those of GFP-FKBP-LBR in figure 6C, for instance. The sentence "reducing the expression of other ER proteins didn't allow for specific labeling" is misleading; again, I see no difference in the specificity of labelling in fig S12C PGK and crCMV and other data using LBR or fragments thereof (the quantified data in S12D-F is for S12B, not S12C).

We cannot directly compare Figure S12C to other Sec61β relocalisation data in the paper (for example in S12B), because in S12C, the MAPPER construct is expressed in these cells, and the level of MAPPER expression (not inducible) may alter the contact site extent and stability. It is for this reason that the quantification in S12E and F is from S12B and not S12C.

In Figure S12C, we see that FKBP-GFP-Sec61β relocalises to areas larger than sites marked by MAPPER; even at very low levels of expression. Whereas in Figure 2, LBR-FKBP-GFP relocalised to labels pre-existing ER-PM contact sites labelled by MAPPER, and not beyond it.

We have not directly compared the relocalisation to different fluorescent protein-tagged Stargazin (Stargazin-mCherry-FRB and in Stargazin-EBFP2-FRB) as it is not anticipated that the fluorescent protein used could affect the relocalisation. Moreover, since LBR is relocalised in a similar pattern to an alternative anchor (SH4-FRB-EBFP2), a peripheral membrane protein, a difference between two more similar anchors is not expected.

It is true that the size of Sec61β clusters appears to decrease with lower expression, however the density of clusters is just as high at low expression as it is with high levels of expression, which indicates labelling is still erroneous. The apparent decrease in size is likely due to the lower fluorescence due to lower expression affecting the way that we classify a cluster from background.

> 2-Explaining why LBR works differently to sec61 appears paramount to generate a useful method. The authors do not agree, so I will illustrate my point. A reporter such as the one described here will induce artifacts because of the high affinity of the FKBP-FRB interaction, and the elasticity of membranes; if more proteins are available to engage in interaction, then new interactions will be formed, which will increase the tethering between the organelles, and thus the surface of contact. The only way to limit artifacts is to reduce the number of proteins that can engage in an interaction. There are two ways of reducing the number of proteins involved in the interaction. First is to reduce the absolute protein level (see point 1), and 2nd is to reduce the availability of the protein for interaction, including (but not limited to), for instance, reducing its ability to diffuse towards contact sites. Now

> what could specifically reduce LBR's availability for interaction?
> According to the authors, the 1st 254 aminoacids only are necessary to do the trick. In these 254 aminoacids we find the transmembrane domain (of course) and a perfectly folded and functional Tudor domain (e.g. PMID:22052904). Tudor domains bind acetylated lysines through pi-stacking, and the aromatics necessary are conserved in LBR. This is likely, together with lamin binding, one reason for the concentration of LBR in the inner nuclear membrane (binding methylated histones in the chromatin). In the cytosol, LBR's Tudor domain might bind other methylated residues, on ribosomes, spliceosomes, Hsp90 and such, or phase separate into nanodomains, as other Tudor proteins have been shown to do (PMID:34115980). This might be what limits the amount of LBR molecules able to engage in contact-site interactions. That might be a very good thing until it becomes a problem. As this is a method paper, it directs future users to leverage a tried a true method for assessing contact sites amounts and localization in a way that is "non-disruptive and does not change ER-MCSs", in this or that experimental conditions. Now "this or that" situation (be it a drug treatment, a genetic alteration, a metabolic or differentiation status) might very well affect the degree of lysine/arginine methylation, the phase separation of tudor domains, chromatin structure, Ribosomal proteins PTMs, etc. which will, in turn change the availability of LBR, and hence the amount of contact site detected by the method. This is why it is much more desirable to know what controls the availability of proteins available to engage in contact sites (e.g. by modulating expression level), rather than relying on an unknown process. Now of course this whole story of tudor domains is invented and might be wrong. The authors might find that a tudor-domain mutant works just as well. But we still wont not know what makes the reporter work, and whether "this or that" situation affect contact sites, or affect the availability of LBR through other mechanisms.

We do not dispute the logic of this argument. In fact, it is what we present in our paper and this is thanks to the Reviewer's input that we have been able to do this effectively. 1) we show that expression levels do not explain the difference and 2) we suggest that there is a molecular explanation in the N-terminal region of LBR. Admittedly, we do not provide a definitive molecular explanation, only some indications, but we maintain that this issue is not “paramount” for researchers to be able to use the method.

We are suggesting the LaBeRling system is used for fast, short-term labelling of MCSs. We have shown that the LBR labels sites pre-labelled by MAPPER and does not expand current contacts or induce new sites over time.

While it is true that we have not identified the exact property of LBR that allows this protein to label MCSs in this way, there are several possible mechanisms that could explain the observed behaviour, in addition to the Tudor domain which Reviewer 1 speculates could be responsible. It is possible that a combination of these mechanisms may occur. Of course, we tried to make further truncations in the Tudor region, but these constructs were not processed correctly within the cell. So although it is unsatisfying, we haven't been able to narrow down the molecular determinant for LaBeRling any further. Even if we established what determines the availability of protein to engage in contact sites, we cannot be sure that there are not other influencing factors (local membrane curvature, composition, diffusivity) which may contribute. Due to this complexity, determining precisely how this works is clearly beyond the scope of this work which is to demonstrate the utility of the method and apply it to an interesting problem.

> 3-The emerin mitochondrial foci (S10) appear no different from the LBR ones (3A). The Lap2b ER-PM foci (S10) appear no different from the LBR ones (6B top, 6C top). This might be related to points 1 and 2 in that these proteins might be in the sweet-spot for expression and availability.

Emerin and LAP2beta clearly show a more complete coating when relocalised to the plasma membrane than seen with LBR.

In addition to expression level and “availability” suggested, the size of the protein and ability to access MCS sterically (emerin and LAP2β are single pass TM proteins, LBR is multi-pass with 8TM domains) provide additional or alternative explanations for the observed differences in behaviours.

Reviewer 2

We thank the Reviewer for the time they took to read and comment on our revised manuscript.

> Concerns have mostly been addressed but I think the authors should elaborate a little bit in the discussion about the potential role of LBR in generating platforms for Ca2+ flux. If researchers want to use this approach as a contact sites sensor, it's important they are aware of likely impact on calcium signaling.

This is an important point, we have added further comment on calcium flux in the discussion:

As a corollary to this, the report of LBR stabilizing ER-MCSs during mitosis by connecting the IP3 receptor in the ER with VDAC2 on mitochondria, thereby regulating calcium flux and energy levels (Zhao et al., 2024), brings a note of caution. LaBeRling mitochondria-ER MCSs during mitosis, may prime sites for complex formation and affect the calcium flux. The formation of this complex is regulated by phosphorylation and so this issue is unlikely to affect LaBeRling in non-dividing cells, nor is it predicted to affect LaBeRling of ER contacts with other organelles.

Reviewer 3

> The authors have done an excellent job of addressing my remaining concerns, and I think that the addition of figureS12, in particular, is an important inclusion to the work. I am happy to support publication of the manuscript and congratulate the authors on their efforts.

We were very pleased that the Reviewer was happy with our revision work and we thank them for their nice comments.

Reviewer 4

> The reviewers have adequately addressed the concerns I raised.

Thank you to the Reviewer. Apologies that you had to look at it again after being happy last time!
